# Examining What We Know in Relation to How We Know It: A Team-Based Reflexivity Model for Rapid Qualitative Health Research

**DOI:** 10.1177/1049732321998062

**Published:** 2021-03-20

**Authors:** Felicia Rankl, Ginger A. Johnson, Cecilia Vindrola-Padros

**Affiliations:** 1 University College London, London, United Kingdom; 2 Nuffield College, University of Oxford, Oxford, United Kingdom

**Keywords:** reflexivity, teamwork, methodology, qualitative, anthropology, health care, qualitative, rapid qualitative research, United Kingdom

## Abstract

Reflexivity constitutes a core component of qualitative research and has been actively integrated into long-term and “lone ranger” approaches to qualitative research. However, its application to team-based approaches and particularly to rapid qualitative team-based approaches continues to lag behind. In this article, we introduce a reflexivity model we developed for teams undertaking rapid qualitative studies. Utilizing our most recent application of this model to a rapid qualitative appraisal of health care workers’ experiences delivering care during the COVID-19 pandemic as a case study, we identify the steps to put this model into practice and its main outcomes. Our application of the model revealed that the team’s practices could be grouped along four dimensions: design assumptions, data collection and analysis processes, multidisciplinary collaboration, and responsible dissemination. Reflexivity can improve the relations within the team and the quality of the research output, if it is implemented as a continuous and iterative process.

## Introduction

Reflexivity, defined as the authors’ critical analysis of the position they occupy throughout the research process and how they participate in the production of knowledge (Pillow, 2003), has been identified as a core component of qualitative research. Reflexivity is based on an iterative process where the researcher takes on a critical account of their “self-location” (with regard to their gender, class, ethnicity, etc.), interests, assumptions, and life experiences and considers how these factors shape their relationship with study participants the research process and, ultimately, the knowledge that is produced (Pillow, 2003; Visweswaran, 1994). Qualitative researchers reliant on long-term and “lone ranger” models, such as ethnography, have actively integrated reflexivity into the research process (LeCompte & Schensul, 1999). However, researchers engaged in other types of qualitative research, particularly team-based approaches, have highlighted the challenges of integrating reflexivity into their research in a meaningful way (Hesse-Biber, 2016; MacQueen & Guest, 2008). Common difficulties include producing a shared understanding among researchers from different backgrounds, creating a collaborative working environment, and maintaining communication across the stages of the research process (Barry et al., 1999; Bikker et al., 2017). These challenges are particularly salient in studies that utilize a team-based rapid qualitative approach, as the need to produce and share findings in a timely and actionable manner can generate additional internal and external pressures (Johnson & Vindrola-Padros, 2017; Vindrola-Padros, 2020; Vindrola-Padros & Vindrola-Padros, 2018).

In this article, we present a team-based reflexivity model we developed for groups undertaking rapid qualitative research. Here, rapid qualitative research is defined as

intensive, team-based qualitative inquiry with a) a focus on the insider’s or emic perspective, b) using multiple sources and triangulation, and c) using iterative data analysis and additional data collection to quickly develop a preliminary understanding of a situation. (Beebe, 2014, p. 3)

Although its actual length of time depends on its particular characteristics, the timeframe of a rapid study should not resemble the timeframe of a nonrapid study (e.g., the data collection process should not exceed 6 months; Johnson & Vindrola-Padros, 2017). Utilizing our most recent application of this model to a rapid qualitative appraisal of the experiences of health care workers (HCWs) delivering care during the COVID-19 pandemic as a case study, we identify the steps used to put this model into practice and its main outcomes.

### Team-Based Reflexivity in Qualitative Research

Team-based qualitative research is common in health research. Having multiple researchers collect and analyze data may improve the rigor of the analysis, as teams can combine their knowledge bases (Barry et al., 1999). In particular, working in multidisciplinary teams offers an opportunity for assumptions to be challenged and research accounts to be strengthened through collective interpretation (Beebe, 2001, 2014). Multidisciplinary teams are not only able to view the same observations from different perspectives, but they are also able to sharpen outputs through a process of continuous probing and clarification (Armstrong & Lowndes, 2018; Bresler et al., 1996; Scales et al., 2008). As a result, the team-based approach can improve the quality and rigor of the methodological design, analysis, and interpretation of a study (Barry et al., 1999). Conducting research as a team also makes fieldwork less lonely and isolated, as team members can provide emotional support to each other (Barry et al., 1999; Bikker et al., 2017). This is particularly important when conducting research on, or during, a health emergency.

However, a team-based approach can also delay research findings given that exploring everyone’s perspectives and achieving consensus on collective interpretation may be time-consuming. Producing a shared understanding may be particularly difficult in the later stages of the research process, as collaborative writing and authorship on publications can cause disagreements (Watts & Jackson, 1998). An alternative approach, working in hierarchical teams, can result in an unwillingness to share ideas and in frustration among team members (Barry et al., 1999; Siltanen et al., 2006). In multidisciplinary teams, different expertise and levels of experience may produce delays, as additional explanations and training may be required to bring the knowledge base of the team to a similar level (Fernald & Duclos, 2005; Gale et al., 2013). Moreover, if data collection and analysis are conducted by different team members, difficulties maintaining communication and consistency in the research process may arise (Bikker et al., 2017).

Both the benefits and challenges of teamwork may be lost in the process of interpretation. Mauthner and Doucet (2008) argue that, in the production of articles and presentations, the multiple perspectives that enriched the team experience may be overlooked. The authors propose reflexivity as an opportunity to reintegrate these voices into the research process and to strengthen the objectives of the team-based approach—namely, to conduct time-sensitive yet methodologically rigorous research.

In qualitative research, reflexivity refers to the process of critical self-reflection on how the product of research is affected by the researchers’ own assumptions and by the process of conducting research (Davies, 2008; Probst & Berenson, 2014). The concept of reflexivity is rooted in feminist and postcolonialist traditions that sought to highlight the unequal and hierarchical nature of researcher–participant relationships and the oppressive nature of the research process itself (Campbell & Wasco, 2000; O’Shaughnessy & Krogman, 2012; Said, 2014). According to this literature, as the effects of the researcher are found in all stages of the research process, reflexivity should constitute a continuous iterative process (Berger, 2015; Buch & Staller, 2007; Finlay, 2002; Gilgun, 2010). Reflexivity requires the researcher to take “two steps back” from the subject of the research. The “first step” is from the observation of the research, and the “second step” is from the reflection of the observation itself (Bourdieu, 2004). Therefore, reflexivity constitutes an intrinsic component of the production of knowledge in qualitative research, serving to enhance both its rigor and quality (Barry et al., 1999). However, most of the time, reflexivity is described as an individual activity by a “lone ranger” ethnographer (Bresler et al., 1996). There is only sparse literature on reflexive practices within research teams (Jarzabkowski et al., 2015). Some notable exceptions have applied reflexivity to their collective research process.

For example, Bresler et al. (1996) utilized extended memos in which each researcher described their relationship to the subject of their study as a reflexive tool. These memos were used as a prompt for discussions within the team around prior beliefs, values, and attitudes. Their reflexive exercise identified methodological disagreements, ethical issues such as confidentiality, and diverging values as the main issues associated with teamwork in ethnography. Barry et al. (1999) used reflexive writing and subsequent group discussions as tools in their assessment of the effectiveness of their workgroup. They found that their interpretations of the data were grounded in their prior beliefs and that group discussions were important for the construction of a shared understanding. Based on their own experience, Barry et al. (1999) advocated for the use of team reflexivity, arguing that it improves both the functioning of a team and the quality of its research output. Scales et al. (2008) reached the same conclusion but employed a different approach to aid reflexivity. They conducted their study in a mental health trust in two stages. First, they engaged in data collection independently before they collectively analyzed their data. Second, they returned to data collection to address contradictions in their interpretations. They argued that team ethnography can create a collective understanding only if team members share their observations, confront inconsistencies in their interpretations, and consider alternative evidence in their discussions. Trust, however, constitutes an important prerequisite for team-based approaches. Similarly, Bikker et al. (2017) assert that successful teamwork requires trust and flexibility. They followed Barry et al. (1999) in employing orienting accounts and subsequent group discussions as reflexive tools. Their findings led them to conclude that constructing a shared understanding and dividing tasks were critical components of conducting ethnography as a team.

### Rapid Qualitative Research and Team-Based Reflexivity

Team-based approaches are frequently used for rapid qualitative research, to the extent that some approaches such as rapid assessment procedures (RAPs), rapid assessment response and evaluation (RARE), and rapid qualitative inquiry (RQI) are defined in relation to team-based work (Beebe, 2014; Brown et al., 2008; Scrimshaw & Hurtado, 1987). Many of these approaches consider teamwork a core component of the research process because several researchers can cover more ground—that is, they can collect a greater volume of data—in a shorter amount of time than a lone researcher (Beebe, 2014; Brown et al., 2008). The breadth and depth of data included can be expanded, as researchers can spread the workload between them (Bikker et al., 2017; Woods et al., 2000). Furthermore, team members with different expertise and perspectives can engage in a continuous process of triangulation such that data are collected and interpreted from different points of view on an ongoing basis (Beebe, 2014).

As mentioned above, reflexivity is important to enhance both the workings of the team and the rigor of its research. However, to our knowledge, no previous studies have shared their experience of reflexivity in rapid qualitative research. A potential limitation of rapid qualitative research, as identified by the literature, is that rapid study designs may not allow researchers to critically analyze their own role in the research process. In fact, reflections on how their own self-location shaped the production of knowledge are notably absent from written accounts (Vindrola-Padros & Vindrola-Padros, 2018). Given the rising importance of both teamwork and rapid qualitative approaches in research, the lack of literature on team-based reflexive practices needs to be addressed. Using our experience researching the perspectives of HCWs delivering care during the COVID-19 pandemic in the United Kingdom, we present a model for team reflexivity in rapid qualitative research.

### Our Rapid Qualitative Research Team

The RREAL team was created with the aim of delivering rapid, relevant, and responsive research with a clear applied focus. It was envisioned by the co-directors—both medical anthropologists with a history of applied research—as a way to contribute to the development of the field of rapid qualitative research. In conducting research in this field, the team also aimed to question long-standing assumptions regarding the quality of rapid qualitative research—namely, that long-term fieldwork was necessary to produce valid qualitative research and that rapid research could not engage with theory and critical perspectives to the same extent (Vindrola-Padros, 2020). In contrast, the team was founded on the idea that the theoretical and methodological approaches upon which qualitative research is built are one of the most effective tools to gain crucial insights into perspectives relevant to public health perspectives (Vindrola-Padros, 2020).

Our team concentrates on three areas of work: health services research, clinical trials, and global health and health emergencies. It is composed of a core team of five researchers from different disciplines and universities, and a graphic designer. The team has additional capacity to expand the size of the team by integrating graduate students or additional part-time researchers on a project-by-project basis. The members of the team are coordinated to work across multiple projects in parallel. The team strives to implement a “flat hierarchy” of organization such that members of the team are given the opportunity to lead studies according to their own research interests. Although team members are supported by the co-directors, they are given the freedom to coordinate their own subgroups of researchers and to set their own study timelines.

Across all projects, our team also seeks to create a collaborative learning environment where more senior researchers can explore new methodologies and junior researchers are empowered. This approach to teamwork became particularly salient during our experience researching the impact of COVID-19, as “lockdown” restrictions necessitated the development of new methodologies for remote-based forms of data collection. During this time, the team also enabled several graduate students—all of whom had to revise their pre-COVID-19-designed thesis proposals—to contribute to the research needs of a new and unknown global threat. Here, the team’s “flat” organizational structure proved particularly important, as it allowed for the equal contribution of ideas and resources from both senior and junior researchers.

To date, researchers affiliated with the team have conducted a wide range of COVID-19-related research on HCW’s experiences delivering care to include the challenges of carrying out rapid qualitative research in the context of a pandemic (Vindrola-Padros et al., 2020); in-depth analyses of HCW’s experience with personal protective equipment (PPE); HCW’s well-being and mental health; the “knock-on” effects of the pandemic on routine care, palliative care, patient recovery, and rehabilitation; how religion shaped interpretations of infectivity and care; and the unique experience of Black, Asian, and Minority Ethnic (BAME) populations. Our study on HCW’s experiences of delivering care during the COVID-19 pandemic was approved by the Health Research Authority (IRAS: 282069) and the R&D offices of the hospitals where studies took place.

To carry out this broad repertoire, the team underwent an expansion, incorporating researchers on a voluntary basis, integrating graduate students who would use the data for their own theses, and developing relationships with other research teams. At one point, the team was made up of 23 researchers from five different universities, some of which were based outside the United Kingdom. At the time when this reflexive exercise was conducted, the ratio of senior to junior researchers was 3:5. The composition of the team and the pressure to deliver findings in a timely manner incentivized us to continuously reflect on and change our practices as well as to regularly examine our internal team dynamics.

## Team-Based Reflexivity Model

**Figure 1.**
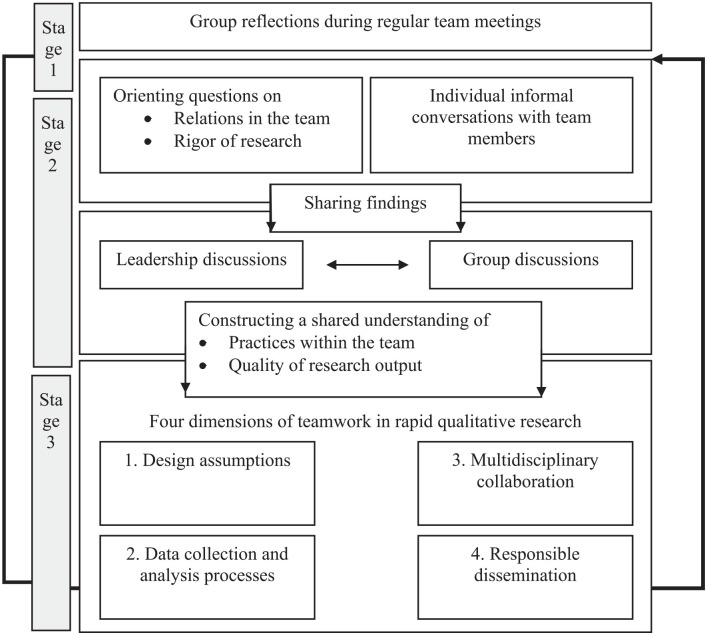
Summary of different steps involved in our model and how these inform each other.

Our reflexivity model followed Braedley’s (2018) approach for creative team reflection and Pillow’s (2003) proposal for the different uses of reflexivity. For a detailed overview over the steps involved in our team-based reflexivity model, see Figure 1. The first stage of the model involved integrating time for team reflections in all of our meetings. In our meeting agendas, we established a dedicated time to talk about ourselves, the work we were carrying out, and any problems we were facing. In particular, we focused on any limitations we could identify in the study and possible strategies to address these limitations. Importantly, these reflections were captured through detailed meeting notes and distributed to the wider team afterward.

The second stage of the model involved informal conversations with team members who contributed to one or more of the above listed COVID-19-related research projects. Orienting questions were developed by the co-directors in collaboration with a junior researcher. The questions were open-ended and covered topics related to experiences working with the team, areas of good practices, and areas for improvement (for a summary, see Table 1). The junior researcher who carried out the conversations with members of the team was selected to guide these discussions because she had spent less time with the team at this point. She guaranteed team members anonymity and removed any identifying details from the findings in advance of sharing them with the rest of the team. By providing team members with orienting questions, we aimed to guide team members, less experienced with reflexivity, in their responses. The recruitment of the team members followed an informed consent process, whereby potential participants were provided with information on the aims of the study and on how the data would be used and stored. They were reminded that participation would be voluntary and that they would remain anonymous. The study was carried out within a larger project focused on the implementation of rapid qualitative research during the COVID-19 pandemic, which was approved by the Health Research Authority (IRAS: 282069).

**Table 1.** Orienting Questions for RREAL Team-Reflexivity.

1. What is the role you currently play within the team? 2. How did you join the team? 3. How long have you been working as part of the team? 4. Why did you join the team? 5. Would you like to have a different, or ongoing role with the team, beyond what you are doing now? Why or why not? 6. Can you describe your overall experience working with the team? How has your experience with the team compared to your expectations when joining? 7. What are some of the things the team does well? 8. What are the areas that need to be improved? 9. Have you felt supported in your work by the wider team? a. If yes, what was the most useful type of support? b. If no, what type of support did you need? 10. If you worked/currently work with other teams, are there any tools/approaches from the team you would implement in these settings/projects? 11. When thinking about the studies implemented by the team, have there been any limitations in their design and implementation? Are there any limitations in their design and implementation that you believe you would not or have not encountered in other long-term projects? 12. If we were to design and implement these studies again, would you do anything differently? Would you have any suggestions to offer for their improvement and/or adaptation? 13. Is there anything else you think we should know that I have not yet asked?

Ultimately, 15 of the 26 team members involved in COVID-19-related research volunteered to participate in the reflexive exercise. Of these 15 members, seven were senior researchers (with 2 or more years of postdoctoral experience) and eight were junior researchers (i.e., MSc and PhD students). Their responses to the orienting questions were recorded and collated in the form of a summary of emerging findings by the junior researcher. This preliminary report was first shared with the two co-directors and discussed at a team leadership meeting. A more developed version of the report was then circulated to the wider team and discussed at a team meeting. It served as a prompt for conversations at subsequent meetings where the identified issues were explored. The notes taken during these collective discussions were compared with the notes from the individual informal conversations with members, and together, formed part of the basis of the team’s future strategy. Our findings revealed that the team’s reflection on their practices can be grouped along four dimensions: design assumptions, data collection and analysis processes, multidisciplinary collaboration, and responsible dissemination. These will be discussed in detail in the following sections.

## Findings and Discussion

### Design Assumptions

Questioning the assumptions that researchers bring to the research process is considered an important aspect of reflexivity. This is because “scientists approach the social world already carrying with them assumptions which partake of the same social world they attempt to study” (Georgaca, 2003, p. 122). We need to acknowledge that the choice of research design and the resulting findings are not objective, as they carry our own assumptions (Mauthner & Doucet, 2003). In our conversations with team members, many noted that our design assumptions had resulted in significant gaps in data collection with regard to the ethnic and professional diversity of HCW interview participants. Indeed, most of the participants interviewed during our initial research efforts were White and higher-grade HCWs from well-funded trusts in London. This bias appears to have been the result of a sampling strategy, which was not comprehensive in the collection of demographic information. As a result, critical details related to the background of participants were not included in the initial interpretation of preliminary findings. One team member remarked that “some things were overlooked in the study design because it was done so quickly.” The fear of missing out on important perspectives in the early stages of the pandemic motivated the team to roll out interviews quickly and to rely mostly on the team’s networks to reach potential participants.

The team-based reflexivity model played an important role in revealing how our assumptions affected the research design. As Braedley (2018) suggested, it allowed us to examine the basis of our choices, as collective discussions of emerging findings drew attention to omissions, elevations, and biases in the data. As we realized that our assumptions critically affected the data we collected and the conclusions we drew, we came to a shared understanding that it was necessary to amend our research design and recruitment strategy. In the conversations held as part of our reflexive exercise, one team member summarized our responsibility to address the omissions in the data as follows:

We tend to amplify certain voices that are already heard, and we don’t always make the effort to dig deeper to hear the voices of people who aren’t heard . . . Researchers need to make the extra effort to hear these voices.

Accordingly, members of the team worked to assess and amend the HCW interview guide to include questions on topics associated with gender, race, and ethnicity. The sampling strategy was expanded to a community care trust, which included more local hospitals than the other two trusts previously recruited, and an active effort was made to recruit non-White and lower-grade HCWs. Additional projects were added to explore the impact of ethnicity and race on the experience of HCWs during the COVID-19 pandemic. In future research, our goal would be to avoid similar flaws in the research design from the beginning. In particular, potential solutions include collecting participants’ demographic information to analyze the representativeness of the sample, relying on snowball-sampling to recruit participants, and conducting interviews in different languages to encourage non-English speakers to participate. Currently, the team is seeking to integrate these approaches into its strategy. For example, we are developing a pool of potential interpreters who can be ready to assist with studies at short notice to avoid delays, even in a health emergency.

During our reflexive conversations, it emerged that both the rapid and collaborative nature of our research enabled us to catch and rectify initially flawed design assumptions. As Baines and Cunningham (2011) note, rapid ethnography is an effective method to enhance data collection and analysis by making them an iterative process. Conducting interviews and interpreting findings concurrently alerted us to how our assumptions affected our research output. Moreover, carrying out data collection and analysis as a team resulted in continuous reflexivity, as we discussed observations and findings with each other. These discussions drew attention to omissions, elevations, and biases in the data (Probst & Berenson, 2014). Finally, the diversity and size of our team played an important role in detecting shortcomings in our assumptions. A smaller, less diverse research team may have overlooked similar biases more easily.

### Data Collection and Analysis Processes

**Figure 2.**
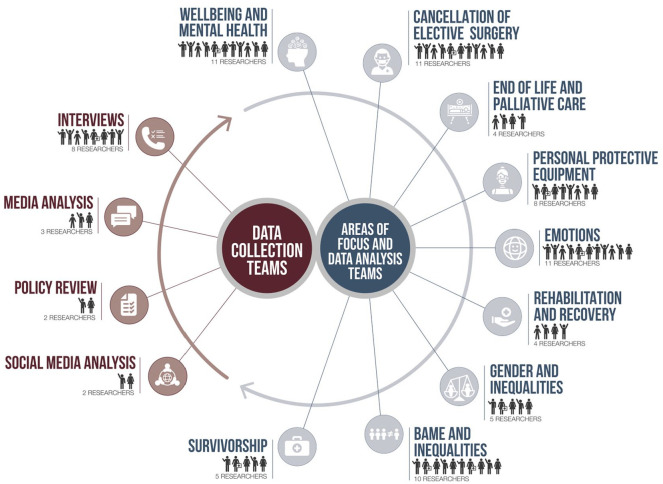
Overview over the organization of the RREAL team.

The scale of our COVID-19-related research demanded separating the wider team into subgroups (for a summary of the team structure, see Figure 2). Our team included four workstreams responsible for data collection—for interviews, (news) media analysis, policy reviews, and social media analysis—and nine workstreams responsible for data analysis. All the workstreams were led by a team member who was ultimately responsible for the output of their respective subgroup. These “leads” were self-selected to coordinate a subgroup according to their research interests. Although there was significant overlap between members working on different workstreams, maintaining communication and consistency between data collection and analysis constituted an important aspect of teamwork. As one team member succinctly put it, “How do you share information about a rapidly changing project across a rapidly changing team when everyone is in different institutions?” Our reflexive conversations revealed that two features were critical to the success of the team-based approach—regular communication and standardized tools.

#### Regular communication

Ellingson (2002, 2003) asserted that interdisciplinary teams in a health care setting can only fully advantage of the breadth of their expertise if they integrate the diversity of their members through effective communication. The same holds for multidisciplinary teams researching health services. Bikker et al. (2017) emphasized the importance of regular teleconferences to maintain communication between their separate workstreams, although their project remained on a much smaller scale. For our study, we established the following meetings via video calls with the different workstreams:

- Team-wide meetings brought members from all workstreams and all institutions together. These meetings were carried out first on a fortnightly and then on a monthly basis. They were used to share emerging findings from data collection and to discuss potential ideas for data analysis.
- Subgroup meetings were held for each workstream in charge of data collection. These were carried out every week during the first few months of the study and were subsequently moved to every fortnight. They ceased when data collection ended.
- Subgroup meetings for each workstream responsible for data analysis were organized by their respective “lead” and were used to make sense of the findings and to discuss emerging themes. These meetings started at different points in time depending on when the project reached the analysis stage. For example, they were first established on a weekly basis to draft a plan and were then spaced out to a fortnightly or monthly basis to provide team members with sufficient time to work on transcription, coding, and writing.
- One-to-one meetings between the team leads and each researcher were held to discuss progress on data collection and/or analysis as well as career development. These were carried out every fortnight at first and later every month. For those graduate students who used the data for their MSc dissertations, these meetings were framed as supervisory meetings.

The organization of the team into subgroups mentioned above meant that the respective workstream meetings could be used to address concerns associated with a particular piece of work. For example, in the meetings organized by the subgroup responsible for interviews, team members were able to share their observations, which often resulted in thought-provoking discussions. As the subgroup included many junior researchers, some of whom were working on their first research project, the ethical considerations of interviewing received particular attention. When interviewing HCWs about difficult topics such as their mental health and personal fears, junior researchers questioned the boundary between objectivity and empathy. They often asked themselves the pertinent question of “where . . . does the responsible ethnographer draw the line and limit participation to remain an ethically responsible observer and reporter?” (Tinney, 2008, pp. 203–204). In our reflexive conversations, many junior researchers noted that meetings played an important role in addressing these ethical questions, as they provided them with the opportunity to discuss their observations and concerns.

Although personal meetings may have encouraged greater mutual understanding (Liggett et al., 1994), remote working conditions during the pandemic clearly prohibited such interactions. Understandably, the absence of face-to-face meetings caused some frustration that emerged in our reflexive conversations. Some researchers missed casual opportunities to build interpersonal relationships and become more familiar with other researchers’ work. As a result, they noted that it was sometimes difficult to understand who was responsible for what parts of the research process. Perhaps, in-person meetings would have allowed for researchers to get to know one another better through non-work-related conversations during breaks. Nevertheless, regular group calls offered an opportunity to construct a shared understanding between team members tasked with data collection and analysis. As a result, they promoted communication between different work streams. Moreover, regular meetings aided reflexivity by incentivizing researchers to regularly examine emerging findings and consider challenges to preconceived assumptions.

#### Standardized tools

One of the challenges of team-based research is ensuring consistency in data collection and analysis across researchers. One strategy that has been developed to address this challenge is the use of standardized tools in the form of tables and guides (Bikker et al., 2017). Our team utilized standardized tools for both data collection and analysis, namely, rapid assessment process (RAP) sheets for the synthesis of data, and framework analysis for the in-depth evaluation of data. These tools enabled us to discuss emerging findings and encouraged us to be consistent in our interpretations. Moreover, both tools were suitable for a multidisciplinary team that included members with limited experience in rapid qualitative research.

In the data collection process, each team member maintained their own RAP sheet, where they organized their notes in line with the categories of the coding framework (for a detailed description of the use of RAP sheets, see Vindrola-Padros et al., 2020). Considering recordings were often hours long and transcription was a slow process, RAP sheets became the primary way of sharing emerging findings with the wider team. Although a standardized system of notetaking existed, some team members nevertheless remarked in our reflexive conversations that differing styles persisted. Indeed, there is an inherent tension in qualitative research between adhering to predefined categories and losing explanatory content (Bikker et al., 2017). This tension may be exacerbated in a team with many graduate students who have yet to learn the level of contextualization that needs to be added to notes for team consumption (MacLeod et al., 2018).

In the data analysis stage, our team relied on framework analysis. First, the categories to code the data were developed in line with the topics of analysis and a codebook was created to ensure consistency in the coding process. Later, additional topics of interviews were identified in interview transcripts or other data sources and added to the framework. The finalized codes were applied to all the data in a spreadsheet (Gale et al., 2013). This method allowed us to analyze data across cases as well as within cases. In the interpretation of findings, the multidisciplinary nature of our team proved particularly useful, as we were able to examine the same data from multiple perspectives. The following section will explore this aspect of teamwork in more detail.

### Multidisciplinary Collaboration

The team approach provides a unique opportunity to unite researchers from different disciplines and with different levels of experience (Choiniere & Struthers, 2018). In fact, having a multidisciplinary team is considered of the main strengths of the teamwork in qualitative research. The research output benefits from diversity in all stages of the research process, as a multidisciplinary team can incorporate different perspectives into the study design and the interpretation of results (Barry et al., 1999). Our team relied on interdisciplinary collaboration and collaboration between senior and junior researchers. Its members were recruited from various backgrounds, including medical anthropology, medical sociology, public health, psychology, and medicine. In addition, local leads at hospitals were asked to act as first points of contact at these interview sites, providing insider knowledge and facilitating recruitment for interviews. The team also comprised a number of graduate students at the start of their career, for whom this project offered a first taste of research work. Importantly, all members were recruited regardless of their age, gender, ethnicity, and educational background.

The team undoubtedly benefited from its diversity in all stages of the research process. For example, researchers with a social science background encouraged the team to experiment with the use of different theoretical frameworks to make sense of data, whereas researchers with clinical expertise provided insight into details regarding care delivery processes in the context of the National Health Service (NHS). During meetings, team members were incentivized to communicate ideas clearly so that researchers with different expertise and limited experience were able to understand them. In turn, researchers from a different academic background were able to challenge our assumptions. As a result, working as a multidisciplinary team compelled all team members to continuously question, evaluate, and justify their positions.

Our research was not only enriched by the perspectives of experienced academics from various backgrounds but also by the contributions of graduate students. Choiniere and Struthers (2018) assert that “fresh eyes can capture what might otherwise be overlooked because of familiarity” (p. 84). For example, graduate students were responsible for amending the interview guide to reflect the situations encountered during interviews. Senior researchers benefited from the fresh perspectives that junior researchers provided, and junior researchers were able to gain practical research experiences (Clark & Drinka, 2000; MacLeod et al., 2018). Indeed, in our reflexive conversations, many graduate students noted that their involvement in the team had exceeded their expectations. They expressed particular appreciation for the opportunity to contribute to research projects, to learn from accomplished academics, and to write articles for the purpose of publication.

However, working in a team with researchers from different disciplinary backgrounds and with different levels of experience is associated with unique challenges. Although graduate students may seek to fulfill the role of professional researchers, they have not yet acquired all the necessary tools to do so (MacLeod et al., 2018). Indeed, in our reflexive conversations, a few junior researchers noted that they felt overwhelmed with the responsibility they were given at times and that they had hoped for more formal support or training opportunities. Before the pandemic, the team had offered general training sessions on rapid qualitative research that graduate students joining the team were invited to. However, as these training sessions had to be discontinued during the COVID-19 pandemic, graduate students joining the team at a later stage were unable to benefit from them. Although peer-to-peer learning and sharing were introduced as an alternative, some graduate students felt that they were not provided with enough formal training or supervision. The realization that junior members of the team did not feel sufficiently supported caused us to discuss different ways of holding training through online sessions, which would enable us to continue to deliver training regardless of the restrictions imposed by a pandemic.

Overall, however, the collaborative nature of our team improved the rigor and quality of our research. It aided reflexive thinking unintendedly, as our different expertise and experience caused us to examine findings from multiple perspectives. The process of clarification and questioning used to construct a shared understanding also led us to reconsider our assumptions and strengthen the quality of our research. However, an open and collaborative working environment is necessary so that a multidisciplinary team can succeed (Abramson & Bronstein, 2004; Vyt, 2008). Woods et al. (2000) assert that many problems arise as a result of undemocratic and hierarchical relations in teams. Team members must “feel confident in speaking openly without fear that their ideas . . . will be criticized, derided, or betrayed” (Scales et al., 2008, p .26). Therefore, our team model was based on a “flat hierarchy” organizational structure whereby the opinions of all researchers are valued and considered regardless of their type of expertise or level of experience. In our reflexive conversations, team members noted that they always felt like they were able to trust each other and to share their honest opinion in discussions. Indeed, Salas et al. (2005) assert that mutual trust in addition to a shared understanding and close communication is critical for successful teamwork. Although nonhierarchical relations between team members enabled a collegial model of working, it was nevertheless important that someone was ultimately responsible for decisions, which could not be resolved through debate. In our team, the co-directors fulfilled this role and settled controversial matters, such as authorship on manuscripts being developed for publication.

### Responsible Dissemination

The ultimate aim of rapid qualitative research is the creation of studies that are both timely and relevant so that findings can be used to inform changes (Vindrola-Padros, 2020). In the research process, relying on teamwork allows us to gather and interpret a greater depth and breadth of data in a shorter amount of time. In health emergencies, such as during the COVID-19 pandemic, it is particularly important that findings are readily available to shape health policy and practices (Vindrola-Padros et al., 2020; Vindrola-Padros & Johnson, 2020). Therefore, after producing rapid research as a team, it remains critical that this research is equally quickly disseminated to a diverse audience. If we rely solely on publications in academic journals, we risk that findings may be limited to a small and selective audience. Considering the review process often takes months as well, we also risk that findings are no longer relevant when they become publicly available. Baines and Gnanayutham (2018) discussed using small booklets as a way to disseminate findings to a diverse audience when they carried out a multisited ethnography in nursing homes. These short, accessible, multiformat books are intended to be easily understandable for the public, media, and policymakers (Baines & Gnanayutham, 2018). Similarly, we utilized preprints and infographics (e.g., “visual abstracts” of our work) to communicate our emerging findings to the academic community and a wider audience. Because representation is not only important in the research process but also in the dissemination of the research output, we ensured that our infographics represented the experiences of a diverse range of HCWs. Whenever we published an article in an academic journal, our visual abstracts served to make the findings accessible to other audiences. In advance of publication, these materials were distributed to the HCWs we interviewed and to organizations informing the epidemic response strategy in the United Kingdom. Rapid dissemination of our findings led to further engagement with our research team, which in turn aided ongoing and potential future collaborative research efforts.

Considering dissemination constituted a central aim of our research, it is unsurprising that it was repeatedly mentioned during our reflexive conversations. Some team members were concerned about the lack of focus on publications in academic journals, perhaps reflecting pressures to “publish or perish” (Raffaeta & Ahlin, 2015). However, other team members supported an alternative approach with a focus on applying findings in practice—for example, by working in close collaboration with hospitals. One team member even suggested taking this one step further, by expanding publications to newspapers and by building relationships with medical associations and nongovernmental organizations. Our reflexive conversations revealed a general feeling that the rapid qualitative work conducted by the team addressed a gap in anthropological research. As a result, team members felt a clear responsibility to disseminate their findings to a wide audience to inform positive changes in policy and practice.

## Conclusion

In this article, we have explored the processes we used to develop a team-based reflexivity model for rapid qualitative research teams. Our use of this model during the implementation of a rapid qualitative appraisal of the experiences of HCWs delivering care during the COVID-19 pandemic allowed us to continuously question our assumptions and improve our research processes. As a result, we were able to identify limitations in our design at an early stage and implement changes to address these limitations. Moreover, we were able to ensure that the voices of researchers with a wide range of expertise and levels of experience were heard. Their concerns were taken into consideration when reformulating approaches, dividing tasks and delegating responsibilities, and delivering support. Overall, both individual and group “self-reflections” were valuable in discerning the different aspects of teamwork and their impact on the ways in which we produced and disseminated evidence to inform changes in policy and practice.

In this article, we have only presented one example of our use of this model, although we are currently implementing it across all of our studies. One of the main limitations of the team-based reflexivity model presented here is that it has only been applied to our research team. Two factors may have aided its implementation in our team, however. First, a predisposition to collaborative working may have enabled the success of the team’s nonhierarchical organizational structure and of the reflexivity model. Barry et al. (1999) found that a willingness to be open with each other and to learn from each other were important prerequisites for their team-based research. Similarly, our team members were receptive to the idea of nonhierarchical approaches to research from the beginning. Their willingness to express their own opinions and accept each other’s positions may not have been solely the product of our organizational structure but at least in part the result of their personality traits (Bell, 2007). Second, although our team members had a diverse range of expertise, many of them had a background in social science, including the co-directors who are both anthropologists. Team member experience in the field meant that they possessed at least some understanding of reflexivity and of its benefits for the rigor of a study. They were also open to the idea of engaging in the kind of self-aware, reflexive thinking that enabled the application of this model to our study. Perhaps, the implementation of the team-based reflexivity model may prove more difficult in teams composed of members with little or no experience in reflexivity. It may also be impeded when team members do not agree on how reflexivity can best aid the quality and the rigor of a study. For example, a large team composed mainly of researchers from a clinical background may find a reflexive exercise time-consuming if they have to construct a shared understanding of reflexivity first. Additional work needs to be carried out to examine research teams’ experiences with this and similar models to assess their adaption to different research contexts, types of teams, and study aims. It should also explore how the composition of a research team affects the implementation of team-based reflexivity models. We hope that our discussion of this team-based reflexivity model can help other researchers engage in critical conversations regarding not only what we know but also how we know it.
